# Analysis of Gas-water Flow Transition Characteristics Based on Multiscale Limited Penetrable Visibility Graph

**DOI:** 10.1038/s41598-020-64021-4

**Published:** 2020-04-27

**Authors:** Jun Han

**Affiliations:** Institute of Educational Technology, School of Education, Capital Normal University, Beijing, 100048 China

**Keywords:** Engineering, Electrical and electronic engineering

## Abstract

It’s a significant challenge for gas-water flow transition characteristics from experimental measurements in the study of multiphase flow systems. The limited penetrable visibility graph has been proved to be an efficient methodology for revealing nonlinear dynamical behaviors of time series. In order to uncovering gas-water flow transitions, gas-water flow experiment was carried out to obtain time series signals related to the transitions of three flow patterns. Then a novel multiscale limited penetrable visibility graph (MLPVG) is used to construct complex networks from many different experimental flow conditions. The multiscale network measures associated with node degree are employed to describe the topological features of the constructed MLPVG. The results show that the multiscale limited penetrable visibility graph can not only effectively characterize flow transition but also yields novel insights into the identification of gas-water flow patterns.

## Introduction

Multiphase flow is a complex fluid phenomenon, widely exists in many fields of industry^1,2^. The study of multiphase flow is of great significance for the progress of science and technology in all walks of life. With the improvement of the requirements of measurement, energy saving and control in the industrial production process, the demand for the identification of multiphase flow characteristics and parameter measurement is becoming more and more urgent, which is also an important aspect of solving the problem of multiphase flow. Multiphase flow is the study of the mixed flow of gaseous, liquid and solid materials. The phase refers to different physical properties or mechanical states of different or the same state of matter, which is defined as the form of existence of matter, i.e. gas, liquid or solid. Multiphase flow often has more than one kind of fluid, that is, the fluid with two or more phase substances flowing at the same time, multiphase flow is also known as multiphase flow.

Multiphase flow includes gas-liquid two-phase flow, gas-solid two-phase flow, liquid-solid two-phase flow, liquid-liquid two-phase flow and oil-gas water three-phase flow^3^. Each specific multiphase flow has many similarities, but it has its own physical characteristics and different flow characteristics. The importance of studying the flow pattern and its transformation lies in that different multiphase flow structures have different flows. Therefore, it is very important for the design and operation of engineering equipment to study and design the multiphase flow structure.

The study of two-phase flow pattern characteristics and flow pattern recognition not only has important industrial application value and academic value, but also can provide important technical support for the safety and automatic production of related industries, the design and operation of management system, the development of two-phase flow measurement instruments. The relationship between flow parameters is different under different flow patterns, which results in the influence of flow patterns on the accuracy of measurement methods. Therefore, in order to realize the measurement of multiphase flow, the influence of flow pattern changes must be considered first.

The multiphase flow in the pipeline presents different flow patterns with different geometric and dynamic characteristics, which can be described by component or phase morphology, but it is difficult to achieve quantitative description, because the flow parameters change with the flow pattern, and the relationship between the forces acting on the fluid and parameters is very complex. These effects include buoyancy, turbulence and surface tension, which are very important in hydrodynamics analysis. They all change with the flow rate, pipe diameter, pipe inclination angle and the properties of each phase fluid^4^. Although the basic equations of fluid are very close, the relationship between the flow parameters in different flow patterns is different, and the accuracy of measurement method in one flow pattern is difficult to achieve in the other. Therefore, if the measurement of multiphase flow is realized, the influence of flow pattern change must be considered first. There are many methods of flow pattern recognition. At the same time, the research of convective pattern is gradually developing towards the formation and transition mechanism of flow pattern, as well as the prediction of convective transformation^5^.

Gas-water two-phase flow transition can be widely observed in many industrial applications. Different to the single-phase flow, two-phase flow usually exhibits many complicated flow structures, known as flow patterns which describe how the two phases are distributed and mixed. The characterization of gas-water flow pattern transition represents a significant challenge. Three typical gas-water flow patterns include bubble flow, slug flow and churn flow. Previous studies indicated that the flow behaviors underlying different flow patterns are distinct and complicated, e.g., slug flow exhibits obviously complex chaotic features. In addition, gas-water flow patterns in different diameter pipes usually present different dynamic flow behaviors. Many efforts have been made to cope with the increasing challenges encountered in the gas-water flows, but the dynamical mechanism governing the gas-water flow pattern transitions is still unclear. The last decade has witnessed a fantastic development of complex network^6–10^, which allows studying complex systems that consists of abundant components interacting with each other in a complicated manner.

Time series data mining methods are usually based on feature representation and similarity measurement, and then mining and analyzing classification, clustering, interest pattern discovery, anomaly pattern discovery and data visualization. To evaluate the state of a complex system based on the time series data is an important research content of time series data. The network of time series data introduces the theory of complex network into the mining and analysis of time series data.

In recent years, the complex network analysis method of time series^9–13^ has become a hot research direction, which has produced many novel research methods. For a review of the research on complex network analysis methods of time series, please refer to the paper^14^ cited in this paper. These methods are increasingly used to solve challenging problems in the areas of climate^15^, brain network^16^, thermoacoustic instability^17^, sunspot series^18^, crude oil prices^19^, traffic flow^20^, and turbulent heated jets system^21^, etc.

In this paper, We have carried out experiments of gas-water two-phase flow and obtained the differential pressure time series signals related to the three flow pattern transitions. Then we use multiscale limited penetrable visibility graph (MLPHVG)^22^ to analyze the signals and infer complex networks from many different flow conditions. The results suggest that the multiscale network measures associated with node degree are very sensitive to the flow pattern transitions, and the multiscale limited penetrable visibility graph allows identifying gas-liquid flow patterns and further enables to characterize flow transitions among three gas-water flow patterns.

### Multiscale limited penetrable visibility graph analysis of time series

Researchers have done a lot of research on complex network analysis of time series and have made great progress in finance, medicine, meteorology and other fields. For example, Costa m. *et al*.^23^ proposed the method of multiscale entropy, which calculated the entropy value of time sequence data on multiple scales, used multiscale entropy value to evaluate the complexity of complex systems, and applied the multiscale entropy algorithm to the evaluation of human physiological systems. Wavelet entropy is the combination of wavelet analysis theory and entropy principle. It gives full play to the advantages of both. It can not only achieve the purpose of information fusion, but also analyze mutation signal more effectively, and can better adapt to the feature extraction of signal. Wavelet entropy can be used to represent the change of signal complexity in time domain, and also to represent many frequency-domain features of signal. Wavelet entropy can reflect the energy distribution information of signal in time domain and frequency domain.

The starting point of visibility graph^11,13,22^ is to use complex network technology to analyze time series data and explore the relationship between time series data structure characteristics and network characteristics.

The visibility graph algorithm is adopted to transform time series data into network, and network topology features are further extracted as the features of complex systems.

More recently, the well-established multiscale horizontal limited penetrable visibility graph^22^ provides an efficient way for characterizing a time series in terms of multiscale analysis and complex network analysis.

Multiscale limited penetrable horizontal visibility graph (MLPHVG)^14,22,23^ is a powerful tool for analyzing time series. The basic idea of MLPVG method can be described as follows: For a time series of length *N*, {*x*(*i*)*, i* = *1, 2, …, N*}, we first define temporal scales in terms of coarse grain process^14,22,23^ and obtain a coarse-grained time series {*y*^s^ (*j*), *j* = 1, 2, …, *N/s*} in the following form

1 $${y}^{s}=\frac{1}{s}\mathop{\sum }\limits_{i=(j-1)s+1}^{js}x(i),1\le j\le N/s$$

where s represents scale factor. Next, we construct limited penetrable visibility graph from the coarse-grained time series {*y*^s^ (*j*), *j* = 1, 2, …, *N/s*}.

**Figure 1:**
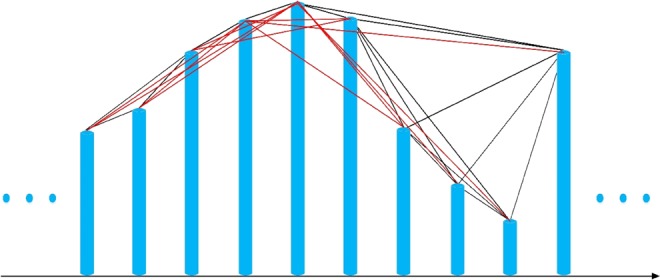
Example of (**a**) the light blue vertical bars represents the measured differential pressure time series signal value and (**b**) an example of LPVG based on the limited penetration distance L of 2, where each node represents the sequential time series data, and the connecting line defines the link of the nodes in the connection diagram.

In Fig. 1, it shows a process example of deriving a a limited penetrable visibility graph from a time series. In the figure, the light blue vertical bars represents the measured differential pressure time series signal value, and the LPVG set to 2 based on the limited penetration distance *L* is exemplified in the figure, each node in the figure represents the sequential time series data, and the connection line defines the link of the nodes in the connection diagram.

In Fig. 1, we illustrate the evolution of LPVG in a continuous time series with a length of 10, and show it in the form of vertical bars and link line.

Considering the evolution process of LPVG in continuous time series, each light blue vertical bar (each value vertex of the time series) is linked to all light blue vertical bars (black lines) that can be seen from the top of the considered bar (black line), so as to obtain the associated visibility graph. In the visibility graph, the line visibility lines between two nodes are connected only when they do not intersect any intermediate data height. At the same time, it means that two nodes (two vertical line vertical bar) can see each other, and then there is a connection between the two nodes.

We can establish the following description and expression of the visibility criteria:

two arbitrary data values (*t*_a_, *E*_*a*_) and (*t*_*b*_, *E*_*b*_) will have visibility, and consequently will become two connected nodes of the associated graph, if any other data (*t*_c_, *E*_*c*)_ placed between them fulfills:

2 $${E}_{c} < {E}_{b}+({E}_{a}-{E}_{b})\frac{{t}_{b}-{t}_{c}}{{t}_{b}-{t}_{a}}$$

In particular, if the limited penetration distance is set to *L*, there is a connection between the two nodes if the number of middle nodes blocking the visibility is not greater than *L*. The red line in Fig. 1 shows that when the limit penetration distance is 2, when VG^11,13^ infers LPVG^13^, a new red line connection is established. Based on the standards and steps described above, LPVG can be derived from coarse-grained time series of different scales to obtain multiscale limited penetration visibility graph (MLPVG)^22^.

The MLPVG^22^ is a type of MLPHVG^14,22,23^ and their basic idea is similar. This method leads to a natural graph-theoretical description of nonlinear systems with qualities in the spirit of symbolic dynamics.

### Multiscale complex network analysis of vertical gas-water flow

The gas-water two-phase flow experiment was conducted in a large diameter vertical pipe. The experimental plan can be described as follows: First we injected the water and gas into a vertical pipe with a fixed water flow velocity, then we gradually increased the gas flow velocity to generate different flow patterns and flow conditions. After this, we changed the water flow velocity and repeat the above procedures. For each generated flow condition, the time series signals can be measured by a sensor. The sampling frequency is 400 Hz and the sampling time is 60 s. Three gas-water flow patterns were observed, including gas-water bubble flow, slug flow and churn flow.

Our method is capable of constructing complex networks at different scales from a time series. Then we use multiscale network measures to analyze the inferred networks. We calculate the average node degree at different scales and then plot it with changing scale factors to characterize the multiscale flow behaviors in the transitions of different flow patterns.

**Figure 2:**
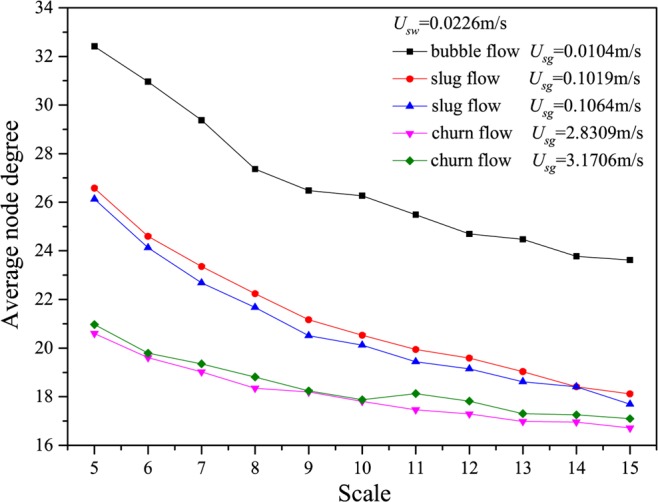
Distribution of the average node degree of MLPVG at different scales for different gas-water flow conditions when *U*_*w*_ = 0.0226 m/s.

**Figure 3:**
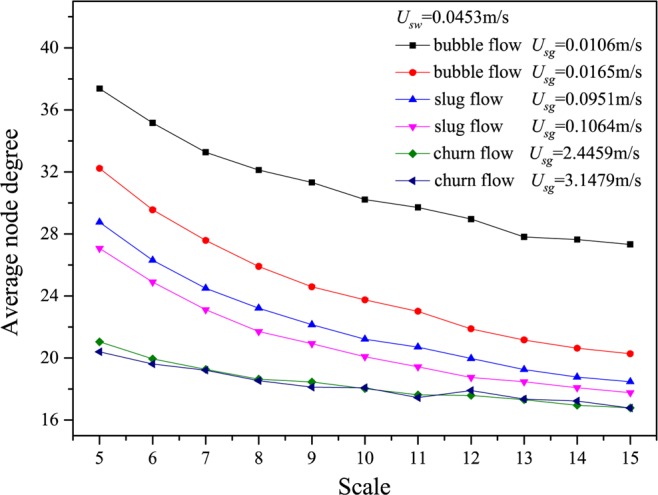
Distribution of the average node degree of MLPVG at different scales for different gas-water flow conditions when *U*_*w*_ = 0.0453 m/s.

**Figure 4:**
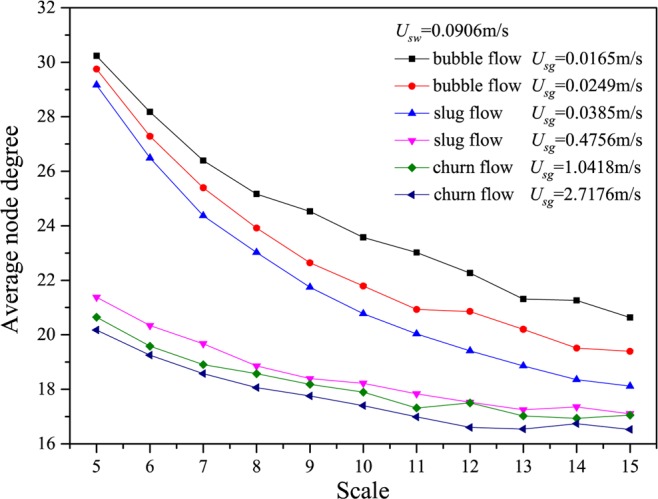
Distribution of the average node degree of MLPVG at different scales for different gas-water flow conditions when *U*_*w*_ = 0.0906 m/s.

**Figure 5:**
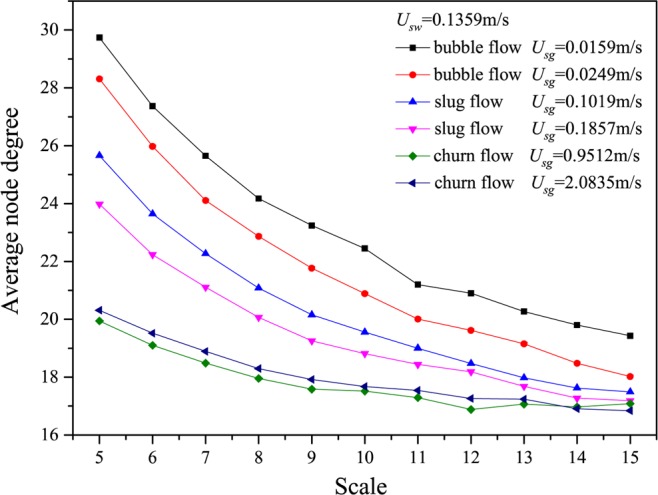
Distribution of the average node degree of MLPVG at different scales for different gas-water flow conditions when *U*_*w*_ = 0.1359 m/s.

**Figure 6:**
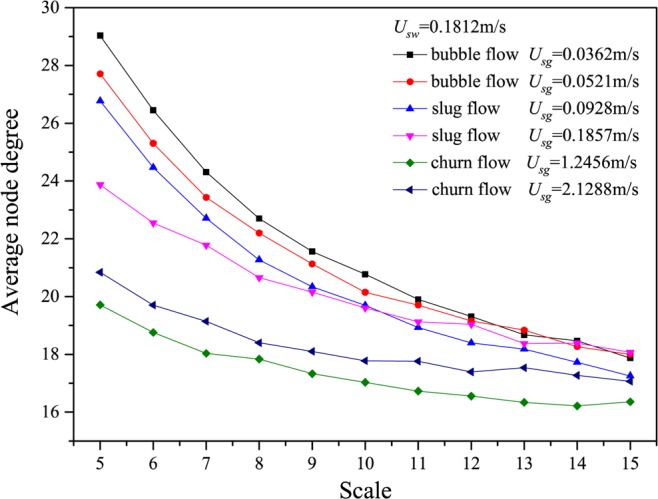
Distribution of the average node degree of MLPVG at different scales for different gas-water flow conditions when *U*_*w*_ = 0.1812m/s.

We show the results calculated from many different flow conditions in Figs. 2–6, in which *U*_*g*_ represents the gas flow velocity and *U*_*w*_ denotes the water flow velocity. As can be seen, the multiscale distributions of average node degree enable to identify three different gas-water flow patterns. Gas-water bubble flow in a large diameter pipe usually occurs at a low gas flow velocity and the typical features lie in the gas phase exists in the form of plenty of discrete bubbles flowing in a water continuum. Because of the stochastic flows of plenty of gas bubbles, the flow behaviors underlying bubble flow is very complex, corresponding to the large average node degree at different scales. With the increase in gas flow velocity, bubble concentration become high and correspondingly bubble coalescence occasionally appears. Consequently, a spherically capped bubble, known as gas slug, is formed with a diameter almost equal to the pipe diameter. Small bubbles randomly flow in the large pipe positions between two slugs. For the gas-water slug flow in a large diameter pipe, the quasi-periodic alternating movements between gas phase and liquid phase are dominant and the chaotic features can be found. In the bubble to slug flow transition, the complexity underlying dynamic flow behaviors becomes lower, leading to the decrease of average node degree at different scales. With a further increase of gas flow rate, the gas-water interface of the larger gas bubble becomes distorted near the nose, but still comparatively smooth in the bottom part of a cylindrical gas bubble. For a high flow velocity, churn flow occurs which can be interpreted as an irregular and oscillatory flow. The chaotic dynamic behavior also exists in the churn flow. The average node degree at different scales also enables to indicate the transition from gas-water slug flow to churn flow.

All these interesting findings suggest that multiscale limited penetrable visibility graph not only can identify different gas-water flow patterns, but also is capable of characterizing flow transitions among gas-water bubble flow, slug flow and churn flow. It should be pointed out that, our work is different to the previous seminal works by Gao *et al*.^13,14^. The previous work^13^ developed a novel multiscale phase-space complex network to analyze multivariate time series and uncovered the flow behavior underlying gas-liquid flow in a small diameter pipe. Their designed four-sector conductance sensor is different to ours. The flow behavior and flow transitions underlying gas-liquid flows in a small diameter and large diameter pipe are very different. Gao *et al*.^14,22^ then proposed a MLPHVG to analyze oil-water flow behaviors and detect epileptic seizure. Our works focus on gas-water flow pattern transitions in a large diameter pipe, which enrich the multiscale analysis and limited penetrable visibility graph theory.

## Discussions

Identifying the gas-water flow transition from experimental measurements constitute a challenging problem of continuous interests. In this paper, we conducted gas-water flow experiment in a large diameter pipe to obtain time series signals related to the transitions of three flow patterns. Then the multiscale complex networks are constructed from many different flow conditions in terms of multiscale limited penetrable visibility graph (MLPVG).

The basic idea of the MLPVG is to define temporal scales in terms of the coarse-grain process and then infer limited penetrable visibility graph from coarse-grained time series for each scale to construct a multiscale complex network. The multiscale network measures associated with node degree are used to assess the topological features of the constructed gas-water flow networks. Our findings suggest that the network measures at different scales are capable of revealing the flow transitions associated with gas flow velocity and water flow velocity. Our analysis also yields novel insights into the flow pattern identification.
